# Development of an RT-LAMP Assay for the Rapid Detection of SFTS Virus

**DOI:** 10.3390/v13040693

**Published:** 2021-04-16

**Authors:** Shiori Sano, Shuetsu Fukushi, Souichi Yamada, Shizuko Harada, Hitomi Kinoshita, Satoko Sugimoto, Tomoki Yoshikawa, Takeshi Kurosu, Yuki Takamatsu, Masayuki Shimojima, Shoichi Toda, Yuka Hamada, Naoki Fujisawa, Takayuki Sugimoto, Masayuki Saijo

**Affiliations:** 1 Eiken Chemical Co., Ltd., 4-19-9 Taito, Taito-ku, Tokyo 110-8408, Japan; Shiori_Sano@eiken.co.jp; 2 Department of Virology 1, National Institute of Infectious Diseases, 1-23-1 Toyama, Shinjuku, Tokyo 162-8640, Japan; syamada@niid.go.jp (S.Y.); shizuko@nih.go.jp (S.H.); knsht@nih.go.jp (H.K.); ssugimo@niid.go.jp (S.S.); ytomoki@nih.go.jp (T.Y.); kurosu@niid.go.jp (T.K.); yukiti@niid.go.jp (Y.T.); shimoji-@nih.go.jp (M.S.); msaijo@nih.go.jp (M.S.); 3 Yamaguchi Prefectural Institute of Public Health and Environment, 2-5-67 Aoi Yamaguchi, Yamaguchi 753-0821, Japan; toda.shouichi@pref.yamaguchi.lg.jp; 4 Kagoshima Prefectural Institute for Environmental Research and Public Health, 11-40, Kinko-cho, Kagoshima City, Kagoshima 892-0836, Japan; hamada-yuka@pref.kagoshima.lg.jp; 5 Shimane Prefectural Institute of Public Health and Environmental Science, 582-1, Nishihamasada-cho, Matsue, Shimane 690-0122, Japan; fujisawa-naoki@pref.shimane.lg.jp; 6 Miyazaki Prefectural Institute for Public Health and Environment, 2-3-2, Gakuenkibanadainishi, Miyazaki City, Miyazaki 889-2155, Japan; sugimoto-takayuki@pref.miyazaki.lg.jp

**Keywords:** SFTS virus, RT-LAMP, simplified method

## Abstract

Detection of severe fever with thrombocytopenia syndrome (SFTS) virus (SFTSV) during the early phase of the disease is important for appropriate treatment, infection control, and prevention of further transmission. The reverse transcription loop-mediated isothermal amplification (RT-LAMP) is a nucleic acid amplification method that amplifies the target sequence under isothermal conditions. Here, we developed an RT-LAMP with a novel primer/probe set targeting a conserved region of the SFTSV L segment after extraction of viral RNA (standard RT-LAMP). Both the Chinese and Japanese SFTSV strains, including various genotypes, were detected by the standard RT-LAMP. We also performed RT-LAMP using the same primer/probe set but without the viral RNA extraction step (called simplified RT-LAMP) and evaluated the diagnostic efficacy. The sensitivity and specificity of the simplified RT-LAMP were 84.9% (45/53) and 89.5% (2/19), respectively. The simplified RT-LAMP can detect SFTSV in human sera containing >10^3.5^ copies/mL viral RNA. The two RT-LAMP positive but quantitative real-time reverse transcription-polymerase chain reaction (RT-PCR) negative samples were positive in the conventional RT-PCR, suggesting that there was no false positive reaction in the RT-LAMP. Both the standard and simplified RT-LAMP are useful for detecting the SFTSV genome in patients during the early phase of the disease.

## 1. Introduction

Severe fever with thrombocytopenia syndrome (SFTS), caused by a novel bunyavirus, is an emerging infectious disease with a high case fatality rate [1]. Common symptoms are fever, general fatigue, and gastrointestinal symptoms; laboratory findings are thrombocytopenia and leukopenia on total blood cell counts. The causative virus is SFTS virus (SFTSV), recently named *Dabie bandavirus*; according to the International Committee on Taxonomy of Viruses, this virus belongs to the genus *Bandavirus* (family *Phenuiviridae*) [2]. Although the virus name was changed to *Dabie bandavirus*, we will use SFTSV here. Humans are infected with SFTSV by a bite from a tick carrying SFTSV, or by direct contact with blood and/or body fluids from an SFTS-infected patient [3,4,5,6,7,8,9]. Recent reports from Japan suggest that patients with SFTS might have been infected by sick cats infected with SFTSV through the cat’s bite or close contact. [10,11,12]. SFTS was first reported in China in 2011 [1], followed by Korea [13], Japan [14], Vietnam [15], and Taiwan [16].

The SFTSV genome consists of three single-stranded negative sense RNA segments, L, M, and S, which encode RNA-dependent RNA polymerase, glycoproteins, and the NSs and nucleoprotein, respectively. Sequence analyses of the viral genome demonstrated genetic diversity among SFTS viruses and reassortants [17,18]. Phylogenetic analyses indicate that either six (A to F) [17] or eight (J1 to J3 and C1 to C5) [18] genotypes exist in East Asia.

Molecular detection methods such as conventional reverse transcription polymerase chain reaction (conventional RT-PCR) and/or quantitative real-time RT-PCR (qRT-PCR) are used widely to diagnose patients with SFTS [19,20] and to investigate SFTSV infection in domestic and wild animals [21,22,23,24]. However, conventional RT-PCR/qRT-PCR requires RNA extraction from blood/serum samples. Although these assays are efficacious for diagnosis of patients suspected of having SFTS, development of a point-of-care (POC) test that amplifies SFTSV RNA is desirable. Reverse transcription-loop-mediated isothermal amplification (RT-LAMP) is a unique nucleic acid amplification method that amplifies the target sequence under isothermal conditions and does not require sophisticated instruments. RT-LAMP is used as a diagnostic tool for patients with specific viral infections such as SFTS [25,26]. To make the RT-LAMP more practical as a POC test, the assay is modified to shorten the time required to complete the reactions [26].

In this study, we developed an RT-LAMP assay based on newly designed primer/probe pairs for detection of the SFTSV genome. Furthermore, we developed a more simplified RT-LAMP from which the RNA extraction process was omitted. We then evaluated its sensitivity and specificity and compared them with those of the qRT-PCR assay.

## 2. Materials and Methods

### 2.1. Primer/Probe for RT-LAMP

**Figure 1:**
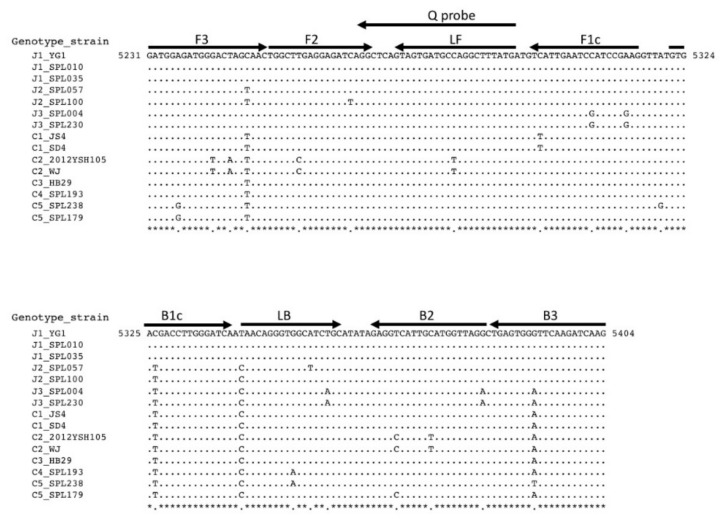
Alignment of SFTSV L segment sequences and positions of the primers used for RT-LAMP assay. The Genbank accession numbers for the L segment sequences of SFTSV strains are as follows, YG1: AB817979; SPL010: AB817983; SPL035: AB817986; SPL057: AB983500; SPL100:AB983519; SPL004:AB817981; SPL230; LC620253, JS4: HQ141604; SD4: HM802202, 2012YSH105: KF711869; WJ: HQ171186, and HB29: HM745930; SPL193; LC620254, SPL238; LC620252, SPL179; LC620255.

The RT-LAMP assay requires a set of six primers: two outer primers (F3 and B3), a forward inner primer (FIP), a backward inner primer (BIP), a forward loop primer (LF), and a backward loop primer (LB). The FIP comprised an F1c sequence connected to F2 sequences. The BIP comprised a B1c sequence connected to B2 sequences. Since the first identification of an SFTS patient in Japan, five genotypes (J1, J2, J3, C4, and C5) based on L segment sequences of SFTSV have been identified [18]. The primers and probe were designed using Eiken Primer Explorer software (Eiken Chemical Co. Ltd., Tokyo, Japan) [27] and were based on the sequences of the SFTSV L segment, which has been identified in Japanese SFTS patients and is available in the GenBank database (Figure 1). Among the six primers, two sets of B3, LB, FIP, and BIP were designed, and used as a mixture in the RT-LAMP reaction. A quenching probe (QProbe) was also designed for accurate detection and monitoring of the quantitative amplification of the target gene [28].

The sequences and positions of the primers and probe are shown in Table 1, along with their binding sites on the SFTSV L segment (Figure 1).

### 2.2. Cell Culture

Vero and Vero E6 cell lines (American Type Culture Collection, Manassas, VA, USA) were maintained in DMEM (Merck KGaA, Darmstadt, Germany) supplemented with 5% fetal bovine serum and 100 U/mL penicillin and 100 μg/mL streptomycin (Thermo Fisher Scientific, Waltham, MA, USA).

### 2.3. Viruses

SFTSV strains YG1, SPL004, SPL010, SPL035, SPL057, SPL087, and SPL100 were isolated from serum samples from Japanese SFTS patients [18]. A Chinese strain of HB29 was kindly provided by De-Xin Li and MiFang Liang (Chinese Center for Disease Control and Prevention). Additionally, strains SPL179, SPL193, SPL230, and SPL238 (isolated from serum samples from Japanese SFTS patients in 2015) were used. Viruses were grown in Vero cells. The genotypes of the SFTSV strains (based on the sequence of the L segment) [18] are shown in Table 2. The SFTSV titer [focus-forming unit (FFU)/mL] was determined in a focus-forming assay, as described previously [29].

Heartland bandavirus, Palma virus, Forecariah virus, Rift valley fever phlebovirus (MP-12 strain), Soft tick bunyavirus, Issyk-Kul virus, Hazara orthonairovirus, Dugbe orthonairovirus, Mobala mammarenavirus, Mopeia mammarenavirus, Argentinian mammarenavirus (Candid#1), Lymphocytic choriomeningitis mammarenavirus, Nipah henipavirus, Zika virus, Severe acute respiratory syndrome (SARS)-related coronavirus, and Middle East respiratory syndrome (MERS)-related coronavirus were grown in Vero or VeroE6 cells. RNA extracted from the supernatants of the virus cultures was used to investigate the cross reactivity of the RT-LAMP assay developed in this study.

### 2.4. Serum Samples

Serum samples collected from SFTS patients (in either the acute or convalescent phase) for diagnostic purposes were selected randomly to compare the RT-LAMP with qRT-PCR [19]. All samples were collected during public general diagnostic surveillance of suspected SFTS. Samples were anonymous.

### 2.5. RNA Extraction

Total RNA was extracted from 200 μL of serum or virus culture supernatant using a High Pure Viral RNA kit (Roche Applied Science, Penzberg, Germany), according to the manufacturer’s protocol. The elution volume for RNA extraction was 50 μL.

### 2.6. Standard and Simplified RT-LAMP Assay

The RT-LAMP assay was performed as previously described [28]. Briefly, the total volume of the reaction mixture was 25 μL, comprising the primers/probe (Table 1), 1.4 mM of deoxynucleoside triphosphates, 0.5% Tween 20, 8 mM MgSO_4_, 30 mM KCl, 20 mM Tricine (pH 8.6), 25 U of Bst DNA polymerase (New England Biolabs, Ipswich, MA, USA), 1 U of avian myeloblastosis virus reverse transcriptase (Roche, Basel, Switzerland), and 5 μL of extracted RNA. Among the six primers, two sets of B3, LB, FIP, and BIP were designed (Table 1), and used as a mixture in the RT-LAMP reaction. The mixture was incubated at 63 °C for 30 min in a LightCycler 480 (Roche) or ESEQuant TS2 tube scanner (Qiagen, Hilden, Germany). For the “standard RT-LAMP” assay, viral RNA purified using the RNA extraction kit was used as the template. For the “simplified RT-LAMP”, the template was serum that had been pretreated as described below without the RNA extraction step (this assay was designed to evaluate detection of the SFTSV genome without a purification step). Serum samples used in the simplified RT-LAMP were pretreated as follows: 5 μL serum was diluted with 40 μL (1:8 dilution) of Loopamp viral RNA extraction solution (Eiken Chemical), followed by heating to 90 °C for 1 min. Five microliters of the pretreated serum sample was used as the template. The simplified RT-LAMP was performed using a 25 μL reaction mixture, as described above. The only difference between the standard RT-LAMP and the simplified RT-LAMP is the procedure used for template preparation.

### 2.7. Quantitative Real-Time RT-PCR and Conventional RT-PCR

The qRT-PCR for detecting SFTSV was performed as described [19]. Briefly, the 25 μL reaction mixture comprised primers/probe specific for the SFVSV nucleoprotein, QuantiTect probe RT-PCR master mix and QuantiTect RT mix (Qiagen), and 5 μL of extracted RNA. The reaction was performed in a Real-time PCR cycler MyGo Pro (IT-IS Life Science, Dublin, Ireland). RNA copy numbers were determined by qRT-PCR using standard synthetic SFTSV S segment RNA [19]. Conventional RT-PCR was also performed as described previously [19]. Briefly, the 25 μL reaction mixture comprised primers specific for the SFVSV nucleoprotein, Superscript III RT/Platinum Taq Mix, Reaction mix (Thermo Fisher Scientific), and 5 μL of extracted RNA. The amplified PCR products were detected by agarose gel electrophoresis.

## 3. Results

### 3.1. Detection Limit in the Standard RT-LAMP Assays

Primers and a QProbe targeting a conserved region of the L segment of SFTSV were designed (Table 1 and Figure 1). Due to primer–template mismatches, two sets of the B3, LB, FIP, and BIP primers were designed to improve the detection limit. The 12 SFTSV strains examined comprised five genotypes (J1, J2, J3, C4, and C5) found in Japanese patients and the C3 genotype (Chinese strain HB29); all were used to evaluate the detection limit of the standard RT-LAMP assay. Viral RNAs were extracted from viral suspensions. Viral RNA (copy numbers were determined by qRT-PCR) were diluted 10-fold and used for the RT-LAMP reaction. As shown in Table 2, RNA from all SFTSV strains was detectable with high sensitivity (10–100 copies/reaction).

### 3.2. Cross Reactivity in the RT-LAMP Assay

RNA extracted from the culture supernatants of viruses belonging to the families *Phenuiviridae*, *Nairoviridae*, *Arenaviridae*, *Paramixoviridae*, *Flaviviridae*, and *Coronaviridae* were used as templates for the standard RT-LAMP. No cross-reaction was detected when using RNA extracted from the supernatants of cells infected with these viruses (Table 3).

### 3.3. Simplified RT-LAMP

Serum samples were mixed with the Loopamp viral RNA extraction solution (ratio 1:8) and then heat-treated at 90 °C for 1 min. We found that addition of 5 μL of the heat-treated mixture to the simplified RT-LAMP reaction mixture (total volume, 25 μL) resulted in optimal performance. The detection limit of the simplified RT-LAMP was evaluated alongside the standard RT-LAMP. Total RNA was extracted from serially diluted viral suspensions containing various genotypes of SFTSV (from 1000 to 1 FFU/mL) and then subjected to standard RT-LAMP. The same viral suspensions were diluted, heat-treated, and subjected to the simplified RT-LAMP. The standard RT-LAMP detected SFTSV RNA in viral suspensions containing 10 or 100 FFU/mL virus. However, the detection limit of the simplified RT-LAMP was about 10 times higher (Table 4).

Next, we examined the sensitivity and specificity of the RT-LAMP assays using serum samples from suspected SFTS cases. Serum samples were diluted and heat-treated as described above, and then tested in the simplified RT-LAMP. At the same time, total RNA extracted from serum samples was subjected to both standard RT-LAMP and qPCR. The results of RT-LAMP and qPCR assays are shown in Figure S1. Among 53 qPCR-positive serum samples, 49 (sensitivity 92.5%) and 45 (sensitivity 84.9%) were positive in the standard RT-LAMP and simplified RT-LAMP, respectively (Table 5). By contrast, among qPCR negative serum samples, two were positive in both the standard RT-LAMP and the simplified RT-LAMP (Table 5). As a result, the specificity of both the standard and the simplified RT-LAMP was 89.5% when compared with the results of qRT-PCR. The two serum samples that were negative in the qPCR were positive in the conventional RT-PCR (Serum ID Y8 and Y10 in Figure S1). This indicates that both the standard and simplified RT-LAMP assays showed 100% specificity.

**Figure 2:**
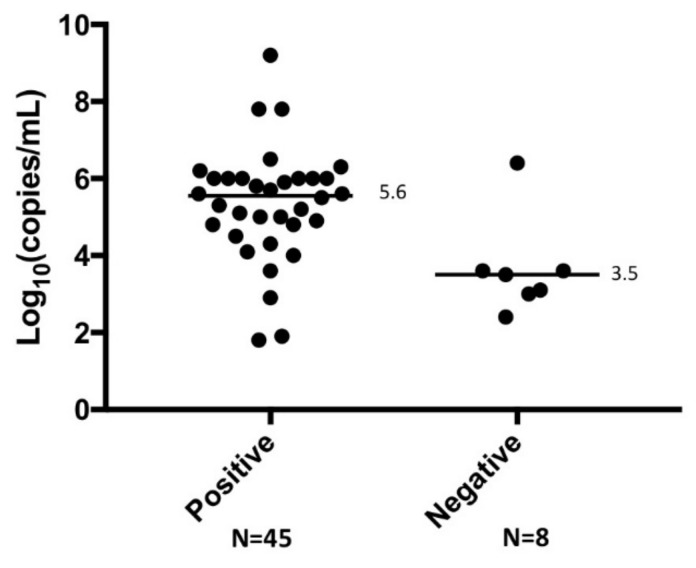
qPCR measurement of RNA copy numbers in serum samples that were positive and negative in the simplified RT-LAMP assay. There was a significant difference (*p* < 0.01) in RNA copy number in the positive and negative samples.

Finally, the RNA copy number determined by qRT-PCR was compared with that in the simplified RT-LAMP-positive and -negative samples (Figure 2). A wide range of SFTSV RNA copies (from 10^1.9^ to 10^9.2^ copies/mL) was detected by the simplified RT-LAMP. There was a significant difference (*p* < 0.01) in the RNA copy number between the simplified RT-LAMP-positive (median value, 10^5.6^ copies/mL) and -negative (median value, 10^3.5^ copies/mL) samples. These data indicate that the simplified RT-LAMP can be used to detect SFTSV in serum samples containing >10^3.5^ copies/mL viral RNA. One serum sample containing 10^6.4^ copies/mL of SFTSV RNA was not detected by the simplified RT-LAMP, probably due to inhibition of the reaction by contaminants or serum components in crude materials.

## 4. Discussion

Detection of specific viral RNA using an RT-LAMP assay takes less than 30 min; this is because the assay amplifies RNA without the need for repeat thermocycling. This method has been used for rapid detection of highly pathogenic hemorrhagic viruses, including Ebola, Marburg, Lassa, and Crimean-Congo hemorrhagic fever virus [30,31,32,33]. RT-LAMP assays for detecting SFTSV have been developed previously [25,26,34,35,36]. Indeed, RT-LAMP assays using primers that target the S segment [34,35] or L segment of the SFTSV genome [25,26,36] detect SFTSV with high sensitivity and specificity. Furthermore, an equipment-free rapid colorimetric visualization method for RT-LAMP has been demonstrated [26]. However, these methods have not been fully evaluated against various SFTSV genotypes, and they still require RNA extraction.

Here, we developed an SFTSV RT-LAMP based on a novel primer/probe set targeting the conserved region of the L segment. Although a set of six primers is usually required for RT-LAMP, we selected two sets of four primers (B3, LB, FIP, and BIP) and a single F3 and LF primer to improve the sensitivity for various SFTSV genotypes, including Chinese and Japanese strains. In a previous study, genetically divergent strains (Musoke and Ravn) of Marburg virus were detected successfully by RT-LAMP using a mixture of two different sets of primers in one tube [31]. Mixing different sets of primers allows detection of multiple SFTSV genotypes. Here, we found that the standard RT-LAMP assay was able to detect SFTSV RNA extracted from 11 virus strains with the J1, J2, J3 C4, or C5 genotypes (all identified in Japanese SFTS patients), as well as the C3 genotype (Chinese strain HB29). Using serially diluted RNA, we demonstrated that the detection limit of the standard RT-LAMP was 10–100 copies/reaction. Although the sensitivity of different viral RNA detection methods cannot be compared directly (due to different methods of preparing RNA from viral suspensions), the sensitivity for detection of SFTSV in this study was similar to that demonstrated by Lee et al. (5 × 10^2^ copies/reaction) [36]; it is also similar to the limits of assays designed to detect MARV, EBOV, and Lassa viruses (20–256 copies/reaction) [30,31,32]. By contrast, the SFTSV detection limit published by Baek et al. (5 × 10^0^ copies reaction) is lower than that in the present study [26]. The reason for the discrepancy in the RT-LAMP detection limit between the study by Baek et al. and others (including our study) is not clear; however, most studies show that the sensitivity of RT-LAMP is about the same as, or slightly lower than, that of qPCR.

Conventional RNA extraction is time-consuming and expensive; as such, this step is a major bottleneck during molecular diagnosis of viral infection. Here, we developed a simplified RT-LAMP using diluted and heat-treated crude serum samples; the assay does not require RNA extraction procedures such as column centrifugation, washing, and elution. The sensitivity of the simplified RT-LAMP was lower than that of the standard RT-LAMP. The higher detection limit of the simplified RT-LAMP can be explained simply. Whereas the simplified RT-LAMP used 5 μL of a 1:8 dilution of serum (equivalent to 0.6 μL of serum/reaction), the standard RT-LAMP used 5 μL of a 50 μL RNA solution extracted from 200 μL of serum (equivalent to 20 μL of serum/reaction). Although the sensitivity of the simplified RT-LAMP is lower than that of the standard RT-LAMP and qRT-PCR, it is worth considering the benefits of the former in terms of speed and simplicity. Furthermore, the serum samples that were negative in the qPCR, but positive in the conventional RT-PCR, were also positive in the simplified and standard RT-LAMP assays, suggesting that the RT-LAMP could detect viral RNA in samples that were possible false-negatives in the qPCR.

One limitation of this study is that we did not test genotypes C1 and C2; this is because these SFTSV genotypes were not available. There was a mismatch in the 3′ terminal region of F1c, which is specific for the C1 genotype (Figure 1); however, because F1c was joined to F2 to form the FIP primer (Table 1), the effect of this mismatch on the function of the FIP primer might be negligible. However, we demonstrated that the RT-LAMP with the novel primer/probe set detected all SFTSV genotypes found so far in Japan.

Importantly, the simplified RT-LAMP can detect SFTSV in serum samples containing >10^3.5^ copies/mL viral RNA (Figure 2). During the early stage of SFTS, which is defined as Days 1–7 after onset of illness, the average viral load in the serum is 10^5^–10^6^ copies/mL [37]. However, between Days 7 and 13 after disease onset, survivors show a decreased viral load in serum, whereas deceased patients have a high serum viral load (up to 10^8^ copies/mL) [37]. It is also demonstrated that most of the SFTSV qPCR positive samples obtained from acute phase have a viremia ranging from 10^4^ to 10^7^ copies/mL, and in the samples from patients who eventually died, the viremia reaches to 10^8^–10^9^ copies/mL [19,38]. These findings suggest that the sensitivity of the simplified RT-LAMP would be sufficient for detection of SFTSV in acute phase serum from SFTS patients.

In summary, we developed an RT-LAMP based on a novel primer/probe set targeting the conserved regions of the SFTSV L segment, and showed that it was able to detect Japanese and Chinese SFTS viruses with high sensitivity and specificity. Furthermore, we developed a simplified RT-LAMP that does not require an RNA extraction step and showed that it also detects SFTSV in patient serum samples. We conclude that the simplified RT-LAMP reduces the time required for detection, obviates the need for complex laboratory equipment, and effectively detects SFTSV from patients’ sera. It could enable wider use as a POC test for SFTS.

## Figures and Tables

**Table 1:** Primer/probe sets used for the SFTSV RT-LAMP assay.

Name	Primer Sequence (5′-3′)	Position	Final Conc. (pmol/Reaction)
SFTS_L_F3	GATGGAGATGGGACTAGCAAC	5231–5251	5
SFTS_L_B3-1	CTTGATCTTGAACCCACTCAG	5384–5404	2.5
SFTS_L_B3-2	CTTGATCTTGAATCCACTCAG	5384–5404	2.5
SFTS_L_FIP-1	CTTCGGATGGATTCAATGAC-TGGCTTGAGGAGATCAGG	5297–5316(F1c) + 5252–5269 (F2)	60
SFTS_L_FIP-2	CTCCGGATCGATTCAATGAC-TGGCTTGAGGAGATCAGG	5297–5316(F1c) + 5252–5269 (F2)	40
SFTS_L_BIP-1	GTGACGACCTTGGGATCAA-CCTAACCATGCAATGACCTC	5322–5339(B1c) + 5364–5384(B2)	60
SFTS_L_BIP-2	GTGATGACCTTGGGATCA-CCTAACCATGCAATGACCTC	5322–5340(B1c) + 5364–5384(B2)	40
SFTS_L_LF	CATAAAGCCTGGCATCACTAC	5274–5294	20
SFTS_L_LB-1	TAACAGGGTGGCATCTGC	5341–5358	10
SFTS_L_LB-2	CAACAGGGTAGCATCTGC	5341–5358	10
SFTS_L_QProbe	CATAAAGCCTGGCATCACTACTGAGCC	5268–5294	1

**Table 2:** Results of three independent standard RT-LAMP assays designed to detect various SFTSV strains. Experiments were performed in triplicate.

Genotype (L Segment)	Strain	Viral RNA Copy Numbers/Reaction		
		100	10	1
J1	YG1	+/+/+	+/+/−	+/−/−
	SPL010	+/+/+	−/−/−	−/−/−
	SPL035	+/+/+	+/+/+	−/−/−
J2	SPL100	+/+/+	+/+/−	−/−/−
	SPL057	+/+/+	+/−/−	−/−/−
J3	SPL004	+/+/+	+/−/−	+/−/−
	SPL230	+/+/+	+/−/−	−/−/−
C3	HB29	+/+/+	+/+/+	−/−/−
C4	SPL193	+/+/+	+/−/−	−/−/−
C5	SPL087	+/+/+	+/−/−	+/−/−
	SPL179	+/+/+	+/+/+	+/+/−
	SPL238	+/+/+	−/−/−	−/−/−

+: detected; −: undetected.

**Table 3:** Cross reactivity of the SFTSV RT-LAMP with viruses belonging to the Phenuiviridae, Nairoviridae, and Arenaviridae families, and with Nipah virus, Zika virus, SARS-related CoV, and MERS-related CoV.

Family	Species	Virus Titer (Log_10_ TCID_50_ or FFU/mL)	Standard RT-LAMP
Phenuiviridae	SFTSV SPL004	8.0	+
	Heartland bandavirus	7.8	−
	Palma virus (Bhanja bandavirus)	7.3	−
	Forecariah virus (Bhanja bandavirus)	7.1	−
	Rift Valley fever phlebovirus (MP−12) *	6.4	−
Nairoviridae	Soft tick bunyavirus (Keterah orthonairovirus)	5.5	−
	Issyk-Kul virus (Keterah orthonairovirus)	5.5	−
	Hazara orthonairovirus	5.9	−
	Dugbe orthonairovirus	6.1	−
Arenaviridae	Mobala mammarenavirus	6.0	−
	Mopeia mammarenavirus	8.4	−
	Argentinian mammarenavirus (Candid#1 **)	6.7	−
	Lymphocytic choriomeningitis mammarenavirus	5.0	−
Paramyxoviridae	Nipah henipavirus	7.6	−
Flaviviridae	Zika virus	6.9	−
Coronaviridae	SARS-related CoV	7.6	−
	MERS-related CoV	7.5	−

+: detected; −: undetected. * Attenuated vaccine strain of Rift Valley fever phlebovirus. ** Attenuated vaccine strain of Junin virus.

**Table 4:** Detection limits of the standard and the simplified RT-LAMP assays. Experiments were performed in triplicate.

Genotype (L Segment)	Strain	LAMP Method	Viral Titer (FFU/mL)			
			1000	100	10	1
J1	SPL010	Standard	+/+/+	+/+/−	−/−/−	−/−/−
		Simplified	+/+/+	+/−/−	−/−/−	−/−/−
J2	SPL100	Standard	+/+/+	+/+/+	+/−/−	−/−/−
		Simplified	+/+/+	−/−/−	−/−/−	−/−/−
J3	SPL230	Standard	+/+/+	+/+/+	+/−/−	−/−/−
		Simplified	+/+/+	+/+/+	−/−/−	−/−/−
C5	SPL238	Standard	+/+/+	+/+/+	+/−/−	−/−/−
		Simplified	+/+/+	+/−/−	−/−/−	−/−/−

+: detected; −: undetected.

**Table 5:** Comparison of SFTSV detection by RT-LAMP and qRT-PCR.

		**qRT-PCR**		
			**Positive (*n* = 53)**	**Negative (*n* = 19)**
LAMP method	Standard	Positive	49	2 *
		Negative	4	17
	Simplified	Positive	45	2 *
		Negative	8	17

* Conventional RT-PCR positive.

## Data Availability

All data relevant to the study are included in the article or uploaded as supplementary information.

## Supplementary Materials

The following are available online at https://www.mdpi.com/article/10.3390/v13040693/s1, Figure S1: Detection of SFTSV in serum samples using the RT-LAMP, qRT-PCR, and conventional RT-PCR assays.
