# Supplementary material for: Development of an RT-LAMP Assay for the Rapid Detection of SFTS Virus
Source: Viruses. 2021 Apr 16;13(4):693. doi: 10.3390/v13040693 (PMC8073756; doi:10.3390/v13040693)
Supplement: Supplementary file 1 [file viruses-13-00693-s001.zip › viruses-1177750-supplementary.pdf]

Figure S1

Serum ID	RT-LAMP		qPCR Log_{10}(viral RNA copy numbers/mL)	Conventional PCR
	Simplified	RNA extraction		
218A1	+	+	9.2	+
107A	+	+	5.6	+
108A	+	+	6.0	+
112A	+	+	4.8	+
192A	+	+	4.5	+
194A	+	+	5.0	+
198A	+	+	7.8	+
106A	+	+	5.5	+
117A2	+	+	7.8	+
M13-17	+	+	4.9	+
M13-22	+	+	5.0	+
M13-24	+	+	4.9	+
M13-35	+	+	6.0	+
M14-01	+	+	6.0	+
M14-10	+	+	6.3	+
M14-11	+	+	6.0	+
M14-25	+	+	6.0	+
K3	+	+	5.1	+
K5	+	+	3.6	+
K8	+	+	5.7	+
K9	+	+	4.5	+
K10	+	+	4.2	+
K11	+	+	4.8	+
K12	+	+	7.1	+
K13	+	+	3.9	+
K14	+	+	5.5	+
K15	+	+	5.6	+
K16	+	+	3.0	+
S1	+	+	6.2	+
S2	+	+	5.3	+
S3	+	+	5.6	+
S4	+	+	6.5	+
S5	+	+	5.9	+
S6	+	+	4.0	+
S7	+	+	5.2	+
S8	+	+	4.1	+
Y1	+	+	3.6	+
Y2	+	+	5.1	+
Y3	+	—	1.8	—
Y4	+	+	5.8	+
Y5	+	+	2.9	+
Y6	+	+	4.3	+
Y7	+	+	4.8	+
Y8	+	+	—	+
Y9	+	+	1.9	+
Y10	+	+	—	+
Y11	+	+	6.0	+

Supplementary Figure S1. Detection of SFTSV in serum samples using the RT-LAMP, qRT-PCR, and conventional RT-PCR assays.

Figure S1
(Continued)

Serum ID	RT-LAMP		qPCR Log_{10}(viral RNA copy numbers/mL)	Conventional PCR
	Simplified	RNA extraction		
186A	—	+	6.4	+
221A	—	+	3.5	+
200A	—	+	3.0	+
109A	—	—	—	—
102A	—	—	—	—
103A	—	—	—	—
M14-02	—	—	—	—
M14-03	—	—	—	—
M14-04	—	—	—	—
M14-05	—	—	—	—
M14-07	—	—	—	—
M13-23	—	—	3.1	+
M13-31	—	+	5.0	+
M14-19	—	—	2.4	+
K1	—	—	—	—
K2	—	—	—	—
K4	—	—	—	—
K6	—	—	—	—
K7	—	—	2.9	+
S9	—	—	—	—
S10	—	—	—	—
S11	—	—	—	—
S12	—	—	—	—
S13	—	+	3.6	—
Y15	—	—	—	—

Supplementary Figure S1 (continued). Detection of SFTSV in serum samples using the RT-LAMP, qRT-PCR, and conventional RT-PCR assays.
